# Sex differences in responses to panicogen CO_2_ inhalation and the role of microglial acid-sensor TDAG8 in female mice

**DOI:** 10.3389/fncel.2026.1865360

**Published:** 2026-08-12

**Authors:** Shawn Lam, Pragnya Sai Priya Munisetti, Rebecca Ahlbrand, Renu Sah, Katherine M. J. McMurray

**Affiliations:** 1 Department of Psychiatry, University of Illinois Chicago, Chicago, IL, United States; 2 Department of Pharmacology and Systems Physiology, University of Cincinnati, Cincinnati, OH, United States; 3 Veterans Affairs Medical Center, Cincinnati, OH, United States

**Keywords:** CO_2_ inhalation, microglia, panic disorder (PD), sex differences, TDAG8

## Abstract

Prior work found microglial acid-sensor T-cell death-associated gene 8 (TDAG8) mediates panic-relevant responses to interoceptive stressor CO_2_ inhalation in male mice. Yet panic disorder is more common in women and panic attack symptoms vary in men and women. Here, we investigated sex differences in CO_2_-evoked behavioral and physiological responses in mice. We also investigated the role of TDAG8 in regulating CO_2_ inhalation-evoked fear-relevant behaviors and hyperventilation, as well as microglial morphology following CO_2_ inhalation in female mice. We found CO_2_ inhalation evoked greater fear-relevant behaviors and hyperventilation in female compared to male mice. Contrary to prior findings in males, we found TDAG8 deletion did not affect fear-relevant defensive responses to CO_2_ inhalation in females. However, TDAG8 deletion significantly attenuated CO_2_-evoked hyperventilation in females. Previous work in male mice showed CO_2_ inhalation induces morphological indicators of microglial activation within circumventricular organ the subfornical organ (SFO) in a TDAG8-dependent manner. Consistent with males, TDAG8 deletion in females attenuated morphological indicators of microglial activation following CO_2_ inhalation within the SFO, but not within another TDAG8-expressing circumventricular organ, the organ vasculosum of the lamina terminalis (OVLT). This suggests a divergence in the role of TDAG8 in mediating behavioral versus physiological responses to panicogen CO_2_ inhalation, though effects on SFO microglia are consistent. Together these data highlight important sex differences in behavioral and physiological responses to panicogen, CO_2_ inhalation. They also highlight the need to consider phenotypic differences when exploring molecular mechanisms and circuitry driving panic-relevant outcomes in males and females.

## Introduction

Panic disorder is a prevalent (∼3% annually) and highly debilitating anxiety disorder characterized by recurrent panic attacks involving episodes of acute fear and physiological symptoms such as hyperventilation (Kessler et al., 2006). Current treatments for panic disorder include selective serotonin reuptake inhibitors (SSRIs) and cognitive psychotherapy, but have limited therapeutic efficacy and a delayed onset of action (Barlow et al., 2000; Kent et al., 2002; Bandelow et al., 2015). Panic phenomena have primarily been investigated in clinical studies that are restricted in their ability to reveal molecular mechanisms. Development of more specific and effective treatments for panic disorder requires knowledge of effector pathways underlying fear and physiological responses that are characteristic of panic disorder.

Mounting evidence suggests that panic disorder is associated with an initial dysfunction in the central homeostatic alarm system wherein normally mild interoceptive stressors trigger heightened fear responses in susceptible individuals (Papp et al., 1993; Colasanti et al., 2012). Physiological homeostasis (i.e., pH balance) is tightly regulated and interoceptive threats to homeostasis induce behavioral, emotional and physiological responses aimed at restoring homeostasis (Craig, 2003; Khalsa et al., 2017). Interestingly, people with panic disorder show heightened responses to interoceptive stressors such as CO_2_ inhalation which induces acid-base imbalance (Fyer et al., 1987; Sanderson and Wetzler, 1990; Papp et al., 1993; Gorman et al., 1997, 2001; Coryell et al., 2006; Leibold et al., 2016; Battaglia, 2017). Rising CO_2_ concentrations produce acidosis and pose a potent interoceptive homeostatic danger signal. Inhalation of low CO_2_ concentrations (5%–10%, 21% oxygen; non-hypoxic) evokes intense fear and hyperventilation that frequently result in panic attacks in susceptible individuals such as those with panic disorder (Gorman et al., 1997, 2001; Bailey et al., 2005). Panic disorder patients report high similarity between CO_2_-induced panic attacks and naturally occurring attacks suggesting CO_2_-inhalation activates effector pathways involved in panic symptoms (Sanderson and Wetzler, 1990; Gorman et al., 2001) and supporting it’s wide use clinically to assess panic disorder vulnerability and pathophysiology (Papp et al., 1993; Rassovsky and Kushner, 2003; Wemmie, 2011; do Amaral et al., 2013). Importantly, in rodents similar CO_2_ inhalation (5%–10%) exposure evokes unconditioned and conditioned fear-like behavior (freezing) and physiological (respiratory, cardiovascular) responses comparable to humans (Ziemann et al., 2009; Johnson et al., 2012; Leibold et al., 2016; Vollmer et al., 2016; McMurray et al., 2019a,^2020^, ^2022^). We have also shown that CO_2_-evoked behavioral and respiratory responses are attenuated by chronic selective serotonin reuptake inhibitor treatment (SSRIs) which are commonly used to treat panic disorder, supporting the predictive validity of this model (McMurray et al., 2019a). Thus, this highly translational cross-species model may have strong value in understanding effector pathways activated by CO_2_ challenge that could ultimately improve treatments.

Recent evidence points to a role for the acid sensing G-protein coupled receptor T-cell death associated gene-8 (TDAG8) and inflammation following CO_2_ inhalation (Vollmer et al., 2016). In male mice, TDAG8 is highly expressed on microglia within sensory circumventricular organs (CVOs) such as the subfornical organ (SFO) and organ vasculosum of the lamina terminalis (OVLT) (Vollmer et al., 2016; McMurray et al., 2019b). The SFO and OVLT have leaky blood brain barriers and are key homeostatic hubs for monitoring brain and plasma milieu (Jeong et al., 2021). Previous work in mice showed CO_2_ evokes inflammatory responses within the SFO, but not the OVLT and that this SFO effect was attenuated in mice with a genetic TDAG8 deletion (TDAG8−/−). The SFO has direct projections to fear and anxiety associated circuitry (e.g., infralimbic cortex, BNST) (Lind et al., 1985; McGuire et al., 2009; Vollmer et al., 2016). TDAG8 deletion and attenuation of SFO inflammation also associated with an attenuation of CO_2_-evoked fear and cardiovascular responses, but not respiratory responses in TDAG8−/− mice (Vollmer et al., 2016).

Importantly, prior work on TDAG8 and CO_2_ inhalation was limited to male mice (Vollmer et al., 2016). Yet, panic disorder in women is ∼2.5x more common than in men, frequently presents with greater symptom severity and has higher relapse rates (Eaton et al., 1994; Sheikh et al., 2002; Yonkers et al., 2003). Men and women also differ in panic symptom presentation, with women more likely to report respiratory symptoms and men more likely to report sweating and pain in the stomach (Sheikh et al., 2002). There is also evidence that women are more sensitive to CO_2_ inhalation (Papp et al., 1997; Nillni et al., 2012; Hall et al., 2025). Differences in the susceptibility to develop panic disorder, symptom presentation and CO_2_ sensitivity point to distinct neurobiological mechanisms contributing to panic disorder within the sexes. Despite this, there have been limited studies addressing underlying mechanisms in women.

Here, we investigated sex differences in behavioral and respiratory responses to panicogen, CO_2_ inhalation. We then determined the effect of TDAG8 deletion on behavioral and respiratory responses to CO_2_ inhalation in female mice. Lastly, we investigated the effect of TDAG8 deletion on microglia morphology following CO_2_ inhalation within the SFO and OVLT.

## Methods

### Animals

**FIGURE 1:**
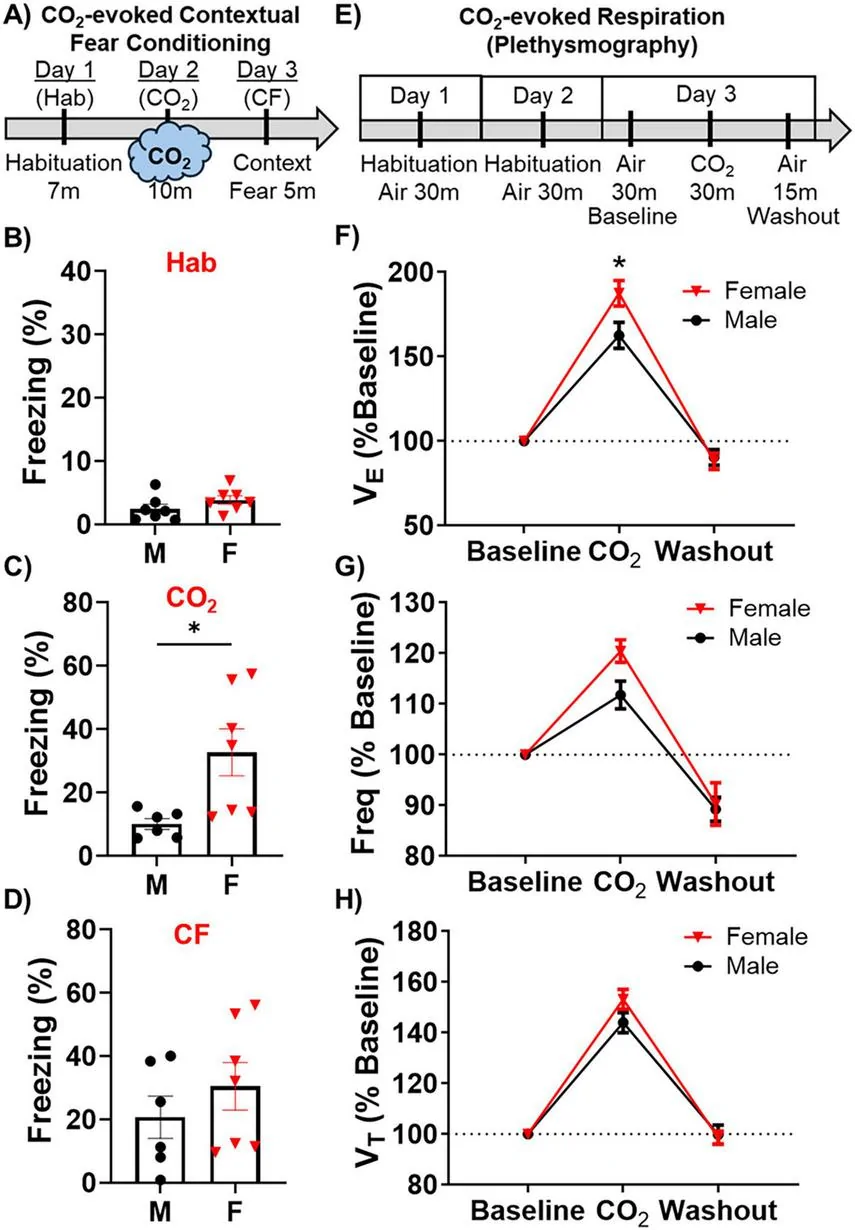
Sex differences in CO_2_-evoked behavior and respiration. **(A)** Timeline of the CO_2_-evoked contextual fear conditioning paradigm. **(B)** Mice did not differ in freezing during habituation to the CO_2_ chamber. **(C)** The following day, there was a significant increase in freezing during CO_2_ inhalation in female compared to male mice. **(D)** Mice did not differ in freezing when returned to the CO_2_ context the following day. **(E)** Timeline of 5% CO_2_-evoked respiration quantification using a plethysmograph. **(F)** Minute ventilation (expressed as a change from baseline initial air exposure on day 3 of the respiratory study) was increased in female compared to male mice during the CO_2_ inhalation phase. However, there were no significant effects on frequency **(G)** or tidal volume **(H)**. *p < 0.05 Hab, habituation; CF, context fear; M, male; F, female; V_E_, minute ventilation; Freq, frequency; V_T_, tidal volume.

**FIGURE 2:**
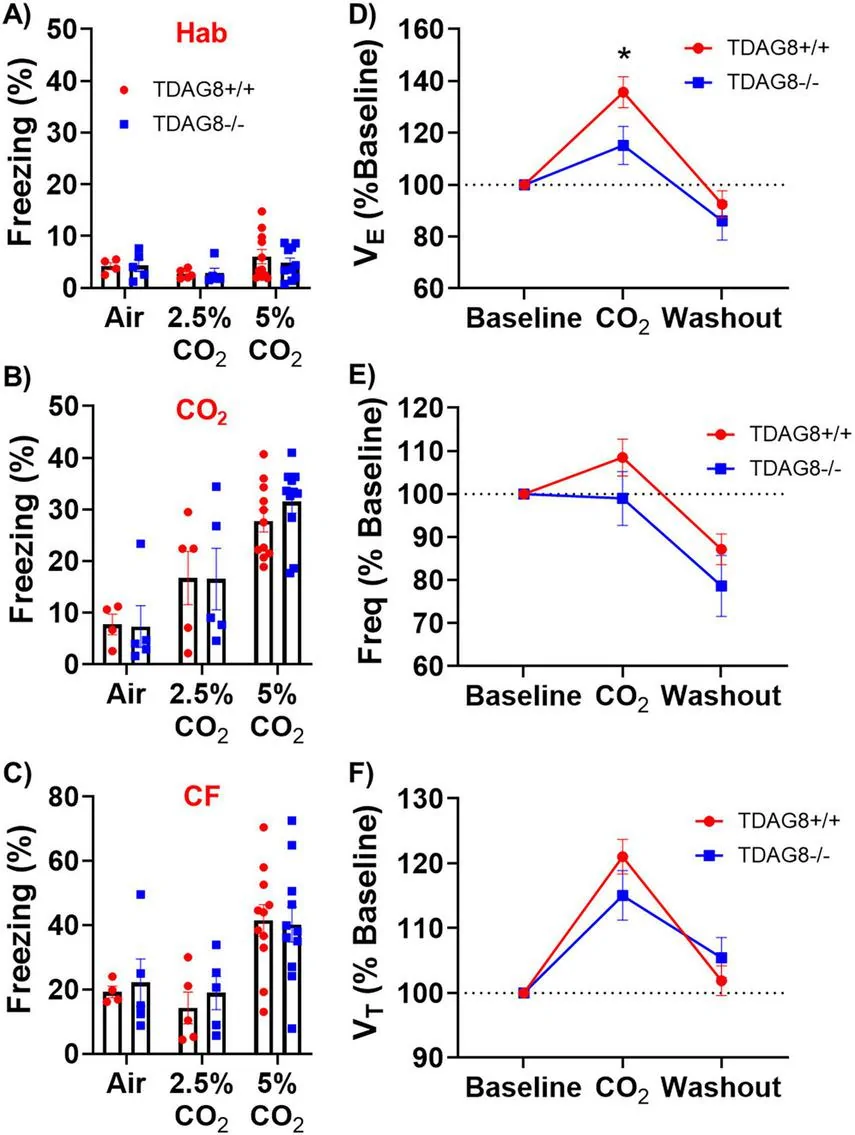
TDAG8 deletion reduces CO_2_-evoked respiration but not fear-relevant behavior in females. TDAG8 deletion (TDAG8–/–) had no effect on fear-relevant freezing behavior during **(A)** habituation (day 1), **(B)** air, 2.5% or 5% CO_2_-inhalation (day 2), or **(C)** during re-exposure to the CO_2_ context (day 3) compared to their wild-type littermates (TDAG8+/+). However, **(D)** TDAG8–/– mice had attenuated minute ventilation (expressed as a change from baseline initial air exposure on day 3 of the respiratory study) compared to TDAG8+/+ littermates during 2.5% CO_2_ inhalation. TDAG–/– and TDAG8+/+ mice did not differ in panel **(E)** frequency nor **(F)** tidal volume. *p < 0.05 Hab, habituation; CF, context fear; V_E_, minute ventilation; Freq, frequency; V_T_, tidal volume.

**FIGURE 3:**
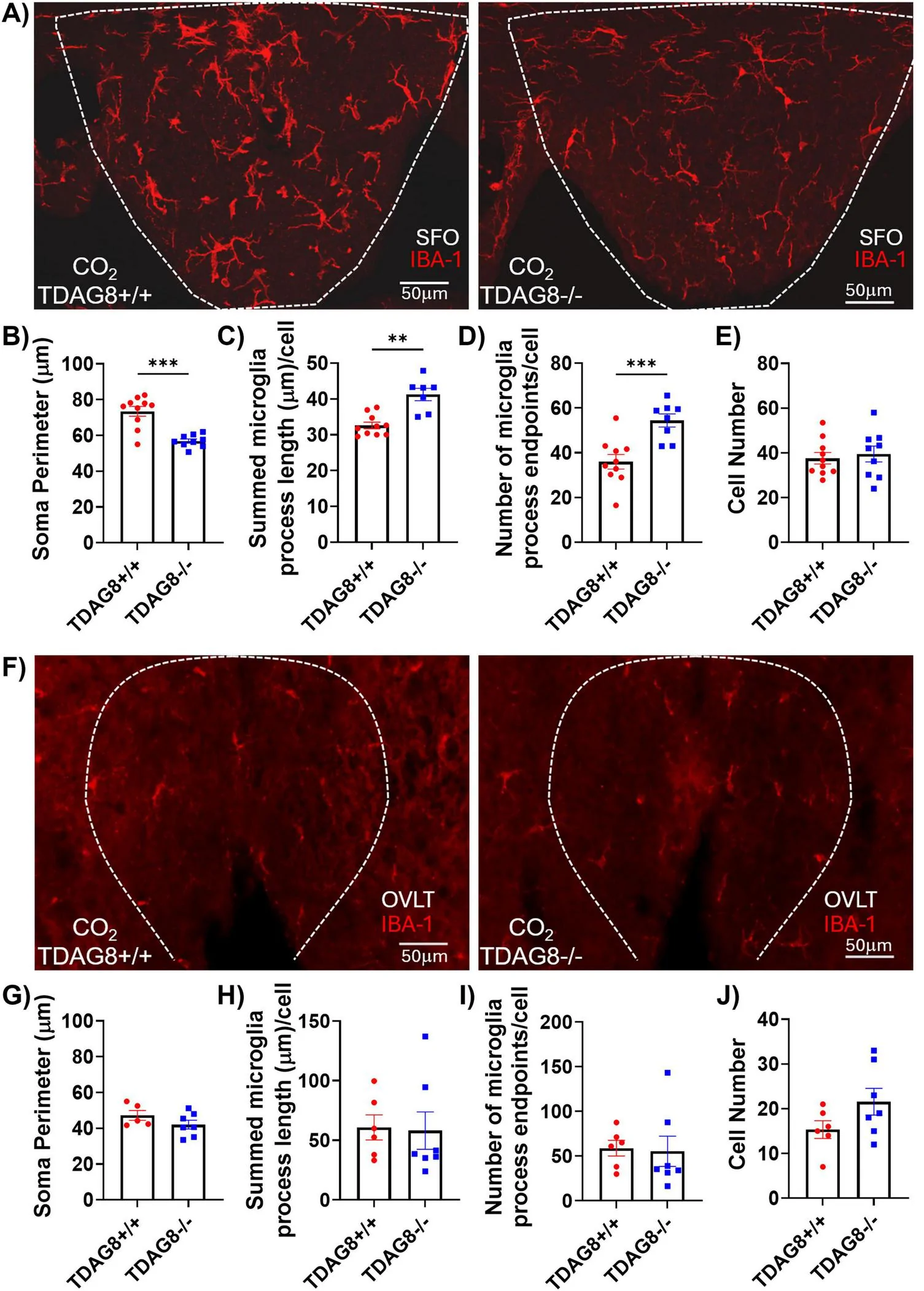
TDAG8 deletion affects microglia morphology in SFO following CO_2_ inhalation. **(A)** Representative images of microglia (IBA-1) in SFO in TDAG8+/+ (left) and TDAG8–/– (right) following CO_2_-inhalation. TDAG8–/– in SFO **(B)** reduced microglia soma perimeter, **(C)** increased process length, and **(D)** increased process endpoints **(E)** without affecting cell number. **(B)** Representative images of microglia (IBA-1) in OVLT in TDAG8+/+ (left) and TDAG8–/– (right) following CO_2_-inhalation. There were no effects of TDAG8–/– in OVLT on **(G)** soma perimeter, **(H)** process length, **(I)** process endpoints, or **(J)** cell number. **P < 0.01, ***p < 0.001.

All studies use mice on a BALB/c background either purchased (wild-type only studies; Envigo, Indianapolis, IN) or bred in house (TDAG8 knockout studies) as follows. TDAG8 knockout mice [Dr. Owen Witte, UCLA) were generated on a BALB/c background as described previously (Radu et al., 2005)]. Male and female mice carrying the wild-type (TDAG8+/+) or knockout (TDAG8−/−) were bred in house, maintained under standard conditions (temperature 23 °C ± 4 °C, 12 h light cycle, ad libitum food/water), group housed (2–4/cage) and tested at 8–16 weeks. Male and female mice are housed in the same room. Studies were approved by the Institutional Animal Care and Use Committees of University of Cincinnati or University of Illinois Chicago and followed the NIH guidelines for the care and use of Laboratory animals. Group sizes were determined based on sample sizes typical in similar prior work (Vollmer et al., 2016; McMurray et al., 2019a,^2022^) and are as follows: In Figure 1, we evaluated seven males and seven females during the CO_2_-evoked behavioral paradigm in wild-type BALB/c mice, and 14 female and 13 males were in the plethysmograph. In Figure 2, we evaluated 4 TDAG8+/+ air-exposed mice, 5 TDAG8−/− air-exposed, 5 TDAG8+/+ 2.5% CO_2_-exposed, 5 TDAG8−/− 2.5% CO_2_-exposed, 11 TDAG8+/+ 5% CO_2_-exposed, and 11 TDAG8−/− 5% CO_2_-exposed mice in the CO_2_-evoked behavioral paradigm, and 5 TDAG8+/+ and 5 TDAG8−/− in the plethysmograph. In Figure 3, microglia were evaluated from 10 TDAG8+/+ and 9 TDAG−/− mice. Due to issues with tissue processing/image quality, this resulted in 9–10 TDAG8+/+ and 8–9 TDAG−/− mice for SFO parameters and 5–6 TDAG8+/+ and 7 TDAG−/− mice for OVLT. Whenever possible, experimenters were blind to genotype (during behavioral/plethysmography studies; while imaging/performing soma perimeter outlining in “Morphological Analysis” below:) and/or automated techniques were used for quantification (CO_2_-evoked behavior, plethysmography, microglial process length/endpoints -Morphological Analysis below). Order of testing and chamber was randomized across groups.

### CO_2_–evoked behavior

CO_2_-evoked unconditioned and conditioned fear-relevant behaviors were measured as described previously by our group (Vollmer et al., 2016; McMurray et al., 2019a). Mice were habituated to a CO_2_ chamber for 7 min. The following day, mice were exposed to air, 2.5% or 5% CO_2_ (21% O_2_ balanced in N_2_) for 10 min. Subjects were then returned to the chamber the day after CO_2_ exposure for 5 min in the absence of CO_2_ for assessment of context-conditioned defensive behaviors. Freezing (complete lack of movement except for respiration; fear-relevant defensive behavior) was scored using the FreezeScan software (CleverSys Inc.).

### CO_2_-evoked ventilatory responses

Whole body plethysmography was used to quantify ventilatory measures in unrestrained, non-anesthetized mice using the Buxco FinePointe system (Data Sciences International, St. Paul, MN), as described previously (McMurray et al., 2019a). Mice were allowed to habituate to the chamber for 2 days (30 min/day) while exposed to room air. On the 3rd day, following placement in the chamber, mice were exposed to air for 30 min to establish baseline respiration. Following 30 min of air exposure, they were then exposed to 2.5% or 5% CO_2_ for 30 min, followed by a 15-min “washout” period of air exposure. Frequency (breaths per minute, BPM), Tidal volume (V_*t*_), and minute ventilation (V_*E*_, the multiplicative product of tidal volume and frequency) were used to quantify ventilation and expressed as a change from baseline air exposure. These were calculated using the Buxco FinePointe system software and excluded data using the Rejection Index (Rinx) feature when Rinx >0 so that analysis only included epochs in which no irregular breathing patterns, apneas, sighs and sniffs (likely related to movement) occurred.

### Immunohistochemistry (IHC)

Ninety minutes following re-exposure to context, mice were perfused transcardially with 4% paraformaldehyde and processed for IHC as previously described (McMurray et al., 2020; Vollmer et al., 2016). Microglia were visualized using primary antibodies against ionized calcium-binding adapter molecule (Iba1; 1:1000 Synaptic Systems cat#234003) and a Cy3 secondary antibody (Cy3 anti-Rb 1:500 Jackson, cat#711-165-152). Immunolabeled sections were imaged at 20x using an AxioImager ZI microscope (Axiocam MRm camera and AxioVision Release 4.6 software; Zeiss).

### Morphological analysis

Methodology to measure morphological changes in microglia in following CO_2_-inhalation was adapted from previous studies (Vollmer et al., 2016; McMurray et al., 2022). Images from Iba1 positive cells in the SFO (−0.10 to −0.82 mm) or OVLT (0.62–0.38 mm) were acquired as Z-stacks and flattened using Image J software (NIH open access). Increased soma perimeter and attenuated microglial branching complexity and process length (de-ramification) were then assessed as parameters associated with microglial activation. The “Freehand line” tool was used outline the soma perimeter of individual microglia and then quantified using the “Analyze and Measure” tool in ImageJ. Soma perimeters from 5 to 10 microglia per slice were quantified, averaged within each subject and these subject averages were used for statistical analysis. For branch length and number of endpoints, images were enhanced to reveal all microglial branches, processed using the “Despeckle” function to remove single pixel background staining within ImageJ, converted into binary images, skeletonized and analyzed using the “Analyze Skeleton” tool in ImageJ. These measures were normalized to the number of cells in each image.

### Statistical analysis

Data are represented as means ± standard error and inferential statistical analyses included students’ unpaired *t*-test, two-way ANOVA or 2-way repeated measures ANOVA as appropriate. Welch’s corrections were applied when distributions had unequal variance. Non-parametric Mann-Whitney tests were applied to distributions failing normality as determined by the Kolmogorov-Smirnov test. For *post hoc* analyses, Tukey tests were applied which correct for multiple comparisons. Grubbs’ tests were performed to determine and remove any outliers (only 1 statistical outlier could be removed from any analyses). Results were considered statistically significant at the *p* < 0.05 level and statistical analyses were performed in Prism, version 11 (GraphPad Software, Inc., La Jolla, CA).

## Results

### Female mice exhibit heightened sensitivity to panicogen CO_2_ inhalation

To investigate sex differences in the spontaneous and conditioned behavioral responses to interoceptive threat, 5% CO_2_ inhalation, male and female wild-type BALB/c mice underwent a CO_2_-contextual fear conditioning paradigm as previously reported by us (Vollmer et al., 2016; McMurray et al., 2019a; Figure 1A). On the first day, mice were allowed to habituate to the CO_2_ chamber and freezing was quantified as a fear-relevant behavior. We found no differences in freezing between male and female mice during habituation [Figure 1B; *t*(12) = 1.414, *p* = 0.183]. The following day, mice returned to the CO_2_ chamber and were exposed to 5% CO_2_ inhalation. We found female mice had significantly increased freezing to CO_2_ inhalation compared to male mice [Figure 1C; Welch-corrected *t*(6.648) = 2.975, *p* = 0.022]. The following day, mice were returned to the CO_2_ chamber in the absence of CO_2_ to evaluate contextually conditioned fear-relevant behaviors. Interestingly, we found no difference in freezing between male and female mice during context re-exposure [Figure 1D; *t*(11) = 0.957, *p* = 0.359].

To investigate sex differences in the physiological responses to interoceptive threat, CO_2_ inhalation, respiration was quantified using a whole-body plethysmograph (Figure 1E) and normalized to the individual’s baseline respiration. For minute ventilation, we found a significant interaction of time and sex [Figure 1F; F(1,25) = 6.605, *p* = 0.017] as well as an overall effect of time [F(1,15) = 260.6, *p* < 0.0001], but no overall effect of sex [F(1,25) = 2.478, *p* = 0.128]. *Post-hoc* tests revealed a significantly heightened minute ventilation in females compared to males during CO_2_ inhalation (*p* < 0.05), but no difference during air washout (*p* > 0.05). For frequency, there was a significant overall effect of time [Figure 1G; F(1,25) = 90.57, *p* < 0.0001], but no significant overall effect of sex [F(1,25) = 2.235, *p* = 0.147] nor interaction of time and sex [F(1,25) = 1.883, *p* = 0.182]. Similarly, for tidal volume there was a significant overall effect of time [Figure 1H; F(1,25) = 259.7, *p* < 0.0001], but no significant overall effect of sex [F(1,25) = 0.975, *p* = 0.333] nor interaction of time and sex [F(1,25) = 2.851, *p* = 0.104].

### TDAG8 regulates respiratory, but not behavioral responses to CO_2_ inhalation in female mice

Previous work found attenuated freezing to CO_2_ inhalation in male TDAG8 knockout (TDAG8−/−) mice (Vollmer et al., 2016). Thus, we next sought to determine the effect of TDAG8−/− on CO_2_ responding in female mice. During habituation to the CO_2_ chamber on day 1, we found no differences in freezing between female wild-type (TDAG8+/+) or TDAG8−/− mice [Figure 2A; genotype: F(1,34) = 0.995, *p* = 0.755; inhalation: F(2,34) = 2.401, *p* = 0.106; interaction: F(2,34) = 0.224, *p* = 0.800]. On day 2, mice were exposed to air, 2.5% CO_2_ or 5% CO_2_. Surprisingly, we found a significant overall effect of inhalation [Figure 2B; F(2,35) = 22.56, *p* < 0.0001], but no effect of genotype [F(1,35) = 0.119, *p* = 0.733] nor genotype by inhalation interaction [F(2,35) = 0.270, *p* = 0.765]. Similarly, when mice were returned to the CO_2_ inhalation context the following day, we found a significant overall effect of inhalation [Figure 2C; F(2,35) = 11.05, *p* = 0.0002], but no effect of genotype [F(1,35) = 0.167, *p* = 0.685] nor genotype by inhalation interaction [F(2,35) = 0.146, *p* = 0.865].

Previous work in males suggested TDAG8 does not affect respiratory responses to CO_2_ inhalation (Vollmer et al., 2016). Given the greater respiratory response to CO_2_ inhalation exhibited in female compared to male mice (Figure 1), we next sought to determine if TDAG8 regulates respiratory responses to CO_2_ in female mice. Female TDAG8+/+ and TDAG8−/− mice were exposed to CO_2_ inhalation as in Figure 1E and respiratory responses were normalized to the individual’s baseline as above. For minute ventilation, we found a significant interaction of time and genotype [Figure 2D; F(1,8) = 9.373, *p* = 0.016] as well as an overall effect of time [F(1,8) = 243.5, *p* < 0.0001], but no overall effect of genotype [F(1,8) = 2.244, *p* = 0.173]. *Post-hoc* tests revealed significantly heightened minute ventilation in TDAG8+/+ compared to TDAG8−/− mice during CO_2_ inhalation (*p* < 0.05), but no difference during air washout (*p* > 0.05). For frequency, there was a significant overall effect of time [Figure 2E; F(1,8) = 138.2, *p* < 0.0001], but no significant overall effect of genotype [F(1,8) = 1.424, *p* = 0.267] nor interaction of time and genotype [F(1,8) = 0.078, *p* = 0.787]. For tidal volume there was a significant interaction of time and genotype [F(1,8) = 21.71, *p* = 0.002] as well as an overall effect of time [Figure 2F; F(1,8) = 198.0, *p* < 0.0001], but no overall effect of genotype [F(1,8) = 0.085, *p* = 0.778]. *Post-hoc* tests did not reveal any significant differences during CO_2_ inhalation or during air washout (*p* > 0.05).

### TDAG8 regulates CO_2_-evoked effects on microglial morphology

As prior work pointed to microglial involvement in CO_2_-evoked fear within circumventricular organ the subfornical organ (SFO) (Vollmer et al., 2016), we assessed microglial morphology which associates with changes in microglial activity following 5% CO_2_-inhalation (Figure 3A). Consistent with previous reports in TDAG8−/− males compared to controls, female TDAG8−/− mice presented with reduced soma perimeters compared to TDAG8+/+ mice [Figure 3B; Welch’s corrected *t*(12.03) = 5.561; *p* < 0.0001]. Conversely, TDAG8−/− presented with increased branch length [Figure 3C; *t*(15) = 4.769; *p* = 0.0002] and endpoints [Figure 3D; *t*(16) = 4.093; *p* = 0.0008]. There were no differences in cell number [Figure 3E; *t*(17) = 0.443; *p* = 0.663]. We also investigated another circumventricular organ which expresses TDAG8, the organum vasculosum of the lamina terminalis (Vollmer et al., 2016) (OVLT; Figure 3F). Similarly consistent with previous reports in males, we saw no differences in soma perimeter [Figure 3G; *t*(10) = 1.363; *p* = 0.203], branch length [Figure 3H; *t*(11) = 0.139; *p* = 0.892], end points [Figure 3I; *t*(11) = 0.172; *p* = 0.866] or cell number [Figure 3J; *t*(11) = 1.675; *p* = 0.122] following CO_2_ inhalation within the OVLT.

## Discussion

Here, we sought to investigate sex differences in the behavioral and physiological responses to CO_2_ inhalation, a highly translational model relevant to panic disorder. We report increased fear-relevant behavior and minute ventilation in response to CO_2_ inhalation in female compared to male mice. We also investigated effects of genetic deletion of microglia acid-sensing receptor TDAG8 on CO_2_-evoked fear-relevant behaviors and hyperventilation within female mice. We report TDAG8 deletion in females reduced minute ventilation but not fear-relevant responses to CO_2_ inhalation. TDAG8 deletion also attenuated morphological effects on microglia following CO_2_ inhalation within the SFO. Collectively, these data suggest female mice show greater CO_2_-sensitivity compared to male mice and suggest a role for TDAG8 in regulating CO_2_-evoked immune and respiratory responses in female mice.

Panic disorder occurs more frequently in women (Sheikh et al., 2002; Yonkers et al., 2003; Kessler et al., 2006) and sex differences in the expression of panic attack symptoms have been well-documented (Sheikh et al., 2002; Yonkers et al., 2003; Hall et al., 2025). Establishing translational models that can be used to identify the underlying mechanisms driving these sex differences is important. Here, we investigated sex differences in responding to interoceptive stressor and panicogen, CO_2_ inhalation. Most neurobiological theories of panic disorder propose an initial dysfunction in the central homeostatic alarm system wherein normally mild interoceptive stressors trigger heightened fear responses in susceptible individuals (Papp et al., 1993; Gorman et al., 2000; Wemmie, 2011; Van Diest, 2019). Initial panic attacks are often spontaneous and accruing data suggests they are associated with increased sensitivity to pH imbalance (Gorman et al., 2000; Wemmie, 2011; Van Diest, 2019). In support of this, CO_2_ inhalation, which results in acidosis, reliably induces panic attacks in panic disorder patients suggesting CO_2_ may activate effector pathways underlying fear responses to interoceptive threat (Fyer et al., 1987; Gorman et al., 1997; Papp et al., 1997; Coryell et al., 2006; Battaglia and Marco, 2013; Leibold et al., 2016; Battaglia, 2017). Thus, understanding effector pathways activated by CO_2_ challenge may ultimately improve treatments.

Interestingly, within people with panic disorder, studies have suggested that women may be more “CO_2_ sensitive” as women show greater symptom severity and vulnerability to panic attack during CO_2_ inhalation (Papp et al., 1997; Nillni et al., 2012; Hall et al., 2025). Exploration of sex differences in rodent studies involving CO_2_ inhalation have been understudied with many reports investigating only male rodents (Leibold et al., 2016; Vollmer et al., 2016; Spiacci et al., 2018; McMurray et al., 2019a,^2020^, ^2022^) or not considering sex as a variable (Ziemann et al., 2009; Taugher et al., 2014). One previous study found no sex differences in CO_2_-evoked freezing within mice on a C57BL/6 background (Ahlbrand et al., 2024). Inconsistencies with this work could be due to differences in the mouse strain and the higher (10%) CO_2_ concentration needed to evoke panic-relevant outcomes in this strain which may engage alternative acid sensing systems like the acid-sensing ion channels (Ziemann et al., 2009). Panic attacks can occur even in healthy individuals as concentrations of CO_2_ raise (Perna et al., 1999; Argyropoulos et al., 2002; Colasanti et al., 2008). It would be interesting in future studies to determine if sex differences in panic-relevant outcomes within BALB/c mice are no longer present at higher CO_2_ concentrations (i.e., 10% CO_2_). Importantly, BALB/c and C57BL/6 mice differ greatly in their innate immune responses (Liu et al., 2002) and stress reactivity (Medina-Saldivar et al., 2024). As we see microglial engagement in BALB/c mice, there could be interesting interactions between sex and immune responses to CO_2_ challenge in BALB/c mice that are not present in C57BL/6 which could also contribute to differences in CO_2_-reactivity in these strains. However, this has not been tested. Our current findings of heightened respiratory and behavioral responses to CO_2_ inhalation in female compared to male mice are consistent with work in rats showing greater minute ventilation in response to CO_2_ inhalation in female compared to male rats (Tenorio-Lopes et al., 2020; Kinkead et al., 2023). Together, these findings are in line with clinical findings on greater CO_2_ sensitivity in women and support the translational relevance of the CO_2_ inhalation model for understanding the pathophysiology of panic disorder.

We previously reported increased TDAG8 expression in panic disorder patients and a strong association of TDAG8 expression with panic symptom severity (Strawn et al., 2017). Previous work in male mice pointed to TDAG8 within the SFO as a key modulator of CO_2_- and acid-evoked fear-relevant behaviors (Vollmer et al., 2016; McMurray et al., 2022; Winter et al., 2022). More particularly, genetic deletion of TDAG8 (TDAG8−/−) attenuated CO_2_ -evoked fear and cardiovascular responses, but not respiratory responses in male mice (Vollmer et al., 2016). Contrary to this prior work in male mice, here we found female mice with a TDAG8 deletion showed no difference in their behavioral responses to CO_2_ inhalation. They did, however, have significantly attenuated minute ventilation compared to wild-type littermates. This may be related to differences in the symptomology of panic attacks that have been reported in clinical studies. One study reporting on findings from the National Comorbidity study found a significantly increased incidence of respiratory symptoms such as difficulty breathing, feeling faint, and feeling smothered during panic attacks in female subjects (Sheikh et al., 2002). Similarly, in response to a CO_2_ rebreathing task women reported greater respiratory effects such as “air hunger” and were more likely to terminate the task (Hall et al., 2025). Overall, these data in female mice are consistent with human data and support a role for TDAG8 in the regulation of panic-relevant outcomes.

While the behavioral and respiratory effects of TDAG8 deletion differed in males and females, the effects of TDAG8 deletion in females on microglia morphology following CO_2_ inhalation are consistent with previous results in males (Vollmer et al., 2016). Microglia are the resident immune cells in the brain which constantly screen their microenvironment to act as sensors of “threat” signals for maintenance of homeostatic balance (Kreutzberg, 1996; Morihata et al., 2000; Hanisch and Kettenmann, 2007). Microglia respond to threats within seconds to minutes progressing to an activated state that can release pro-inflammatory cytokines for downstream responses (Morihata et al., 2000; Hanisch and Kettenmann, 2007). In male mice, previous work showed CO_2_-evoked increases in morphological indicators of microglial activation within the SFO including increased soma perimeter, and reduced microglial process length and endpoints which were attenuated within TDAG8−/− mice. As in male mice, we found that TDAG8 deletion in females reduced microglia soma perimeter, and increased microglial process length and endpoints compared to TDAG8+/+ mice within the SFO, but not OVLT. In future studies using female mice, it will be important to confirm TDAG8 expression on microglia, and more specifically assess whether CO_2_ inhalation induces microglia morphology effects within the SFO. Additionally, future studies should determine the functional consequences of these morphological effects. Traditionally, these morphological changes in microglia (increased soma perimeter/reduced process length/branching) were associated with a more “activated” or neuroinflammatory phenotype that can lead to strong pro-inflammatory cytokine release and phagocytosis (Woodburn et al., 2021). However, more recent work on the effects of stress on microglia morphology and function have suggested these stress-associated effects may have a more homeostatic function affecting neuron-microglia signaling or synaptic level remodeling for example when cytokine release is more moderate (Woodburn et al., 2021; Vidal-Itriago et al., 2022). Future studies will need to determine if the effects here on microglia morphology are indicative of a more pro-inflammatory or homeostatic response.

Studies in male mice have suggested that CO_2_ inhalation activates TDAG8 acid sensors on microglia within the SFO, increasing IL-1β and activating the IL-1 receptor and subsequent effector pathways that result in fear-relevant behaviors associated with panic attack (Vollmer et al., 2016; McMurray et al., 2019a, 2022). Elevated pro-inflammatory cytokines have been reported in panic disorder and panic disorder is often comorbid with pro-inflammatory conditions such as asthma and arthritis, although a mechanistic link between panic disorder and neuroinflammation is yet to be established (Perna et al., 1997; Lavoie et al., 2005). Collectively, these data support a role for inflammatory pathways in panic pathophysiology. However, it will be important it future studies to determine if this same TDAG8-dependent signaling pathway attenuates panic-relevant outcomes, particularly hyperventilation, in females. Additionally, it will be important to determine if these effects are more indicative of more truly inflammatory (large cytokine release/signaling) or homeostatic (moderate/low cytokine release/signaling) microglial response.

An important limitation of this work is that male and female TDAG8 +/+ and TDAG8−/− mice were not directly compared. Nonetheless, it is interesting that the pattern of effect of TDAG8 deletion on microglia morphology within the SFO in female mice was consistent with previous work in male mice (Vollmer et al., 2016), while there were distinct effects on behavioral (previous effect in males, none in females) and physiological responses (previously, no effect seen on respiration in males, effect in females). The SFO has projections to effector sites regulating fear behavior and autonomic responses, such as the infralimbic cortex, hypothalamus, bed nucleus of stria terminalis (BNST), periaqueductal grey (PAG) and brainstem medullary sites (Johnson and Gross, 1993; Shekhar and Keim, 1997). It is possible, therefore, that projections out of the SFO may be differently affected in male and female mice. It will be important that future studies investigating effects of SFO TDAG8 and its downstream effectors/projections on CO_2_ responding include both sexes to allow for more direct comparisons.

Notably, the SFO is an important region for regulating homeostatic responses to circulating hormones throughout the estrous/menstrual cycle and estradiol has previously been shown to inhibit microglial activation (Dimayuga et al., 2005; Donadio et al., 2005; Ciriello and Roder, 2013; Barreto et al., 2014). Mounting evidence suggests hormonal cycling may modulate fear and respiratory pathways in panic disorder patients. Clinical studies have indicated sensitivity to CO_2_ inhalation may vary throughout the menstrual cycle (Perna et al., 1995; Nillni et al., 2011, 2012) and that panic disorder symptoms may be exacerbated during the premenstrual period (Breier et al., 1986; Harrison et al., 1989; Kaspi et al., 1994). A limitation of the work presented here is that estrous cycle was not evaluated. In Figure 1C, there was a significant increase in variance in female compared to male freezing during CO_2_ inhalation, necessitating a Welch’s corrected *t*-test to evaluate differences in group mean. It is possible this is an estrous-dependent effect, but this was not tested. It will be important in future studies to investigate the role of the estrous cycle in driving CO_2_ sensitivity in rodents. It will also be important to determine if CO_2_-evoked immune responses and hormones within the SFO interact to mediate sex differences in downstream signaling outside the SFO.

Differences in the susceptibility to develop panic disorder, symptom presentation and the effect of menstrual cycle on CO_2_ sensitivity point to distinct neurobiological mechanisms contributing to panic disorder within each sex, yet most previous work has focused on males. Here, we report increased CO_2_-sensitivity in female compared to male mice consistent with clinical studies in women. Differences in sensitivity to interoceptive stressors may contribute to the increased incidence of panic disorder in women and translational models probing interoceptive stress sensitivity like CO_2_ inhalation could lead to the identification of novel molecular mechanisms and neurocircuits driving these differences. Additionally, despite the distinct sex effects on behavior and respiratory responses to CO_2_ inhalation, these data collectively support microglial acid-sensor TDAG8 within SFO as an important detector of interoceptive fear-evoking stimuli such as CO_2_-evoked acidosis that mediates panic-relevant outcomes.

## Data Availability

The raw data supporting the conclusions of this article will be made available by the authors, without undue reservation.
